# Optimized Fractional-Order Extended Kalman Filtering for IMU-Based Attitude Estimation Using the Hippopotamus Algorithm

**DOI:** 10.3390/s25226942

**Published:** 2025-11-13

**Authors:** Xiaoping Yang, Gangwang Lin, Jianqi Wang, Lieping Zhang, Jianhui Wen, Xiaoxia Li

**Affiliations:** 1 College of Physics and Electronic Information Engineering, Guilin University of Technology, Guilin 541004, China; gutyxp@126.com; 2 Guangxi Engineering Research Center of Optoelectronic Information and Intelligent Communication, Guilin 541004, China; 3 Guangxi Key Laboratory of Embedded Technology and Intelligent System, Guilin University of Technology, Guilin 541004, China; 4 College of Computer Science and Engineering, Guilin University of Technology, Guilin 541004, China; 3192052051720@glut.edu.cn; 5 School of Aeronautics and Astronautics, Guilin University of Aerospace Technology, Guilin 541004, China; wangjianqi@guat.edu.cn (J.W.); zlp@guat.edu.cn (L.Z.); 6 Guilin Ecological Environment Monitoring Center of Guangxi Zhuang Autonomous Region, Guilin 541199, China; wenjianhui0702@163.com; 7 Guilin Urban Planning and Design Institute, Guilin 541002, China

**Keywords:** fractional-order extended Kalman filter, Hippopotamus Optimization, attitude estimation, parameter optimization, swarm intelligence

## Abstract

The performance of the Fractional-order Extended Kalman Filter (FEKF) is often constrained by the manual tuning of its fractional-order parameter. This paper proposes HO-FEKF, a novel framework that integrates the Hippopotamus Optimization (HO) algorithm to automate and intelligently determine this optimal parameter. Central to our approach is a hierarchical optimization strategy that efficiently minimizes attitude estimation error. In addition to this automated tuning, we enhance the core FEKF model by improving its handling of nonlinear system dynamics, including the effects of time discretization on the Jacobian, cross-factor interactions, and the use of a sliding residual window. We validated our method on both a public benchmark and a custom-collected dataset. Results show that our improved FEKF surpasses the traditional version, and the complete HO-FEKF framework significantly outperforms approaches based on other optimization algorithms (genetic algorithm GA, grey wolf optimizer GWO, Harris hawks optimizer HHO, and HiPPO-LegS algorithm) combined with FEKF. These findings confirm the practical potential of HO-FEKF for achieving adaptive, high-accuracy attitude estimation in real-world sensor fusion scenarios.

## 1. Introduction

Attitude prediction and estimation constitute a core issue in modern navigation systems and are widely applied in UAV flight control, missile guidance, and virtual reality devices [1,2]. The accuracy of attitude angles directly affects the system’s stability and performance. In practical applications, due to noise, drift, and external disturbances affecting inertial sensors, traditional filtering algorithms often suffer from reduced accuracy and instability in attitude estimation [3]. A particularly challenging yet common research background involves the navigation of autonomous systems, such as UAVs, in ‘magnetically denied environments’, including indoor factories, urban canyons, or areas with significant metallic infrastructure. In these environments, ambient magnetic fields are severely distorted by electromagnetic interference from power lines, electric motors, and ferrous structures, rendering magnetometers—the primary sensor for providing an absolute heading reference—highly unreliable. Consequently, accurately estimating the yaw angle becomes infeasible. However, for many critical tasks in these scenarios, such as maintaining platform stability, balancing, and immediate trajectory control, the precise and rapid estimation of roll and pitch angles is of paramount importance, while the absolute yaw angle is often non-essential. Therefore, this study focuses specifically on enhancing the accuracy and robustness of roll and pitch estimation.

To address this challenge, the Kalman Filter (KF) [4], as an optimal state estimation method, provides the theoretical foundation for such tasks. Building on this, the Extended Kalman Filter (EKF) [5], as its application to nonlinear systems, has become the mainstream approach in Attitude and Heading Reference Systems. However, EKF assumes that the system dynamics are governed by integer-order differential equations, which limits its ability to effectively capture the long-memory effects inherent in complex dynamic systems. This limitation becomes especially prominent in high-dynamic scenarios where the system state is significantly influenced by its historical behavior [6].

To address this limitation, the introduction of fractional-order calculus into the Kalman filtering framework can effectively enhance the modeling capability for historical dependencies and dynamic nonlinearities. The FEKF incorporates fractional derivatives and fractional residuals to refine the state prediction and covariance update processes, thereby significantly improving its adaptability to noise and nonlinear dynamics [7]. In recent years, fractional-order calculus has gained widespread attention in the fields of control theory, signal processing, and system identification [8,9,10].

However, determining the parameters of the Kalman filter has long been a challenging research problem. In 2023, Wondosen Assefinew et al. addressed the difficulty in tuning the Q (process noise covariance) and R (measurement noise covariance) matrices in EKF for UAV three-dimensional attitude estimation by proposing a Bayesian optimization algorithm based on innovation white-noise consistency analysis to automatically identify the optimal Q and R values [11]. Standard Kalman filter theory, and by extension the optimization methods designed for it, relies on the Markov property—the assumption that a system’s future state depends only on its present state. Methods like the Bayesian optimization proposed by Wondosen et al., which use innovation white-noise consistency analysis, are explicitly designed around this property. They assume that for an optimal filter, the innovation sequence (the difference between the measurement and the prediction) must be a zero-mean, uncorrelated white noise process. However, the integral nature of the fractional derivative means the system’s state is a weighted sum of its entire history. This inherent memory directly violates the Markovian assumption, introducing temporal correlations into the innovation sequence that these methods would misinterpret as poor tuning of Q and R, rather than as a fundamental property of the system’s dynamics.

In the same year, Xue-Bo Jin et al. introduced a parameter-free Kalman filter state estimation method based on attention mechanism learning. This approach employs a Transformer encoder and an LSTM network to extract dynamic features, and utilizes the EM algorithm to estimate system model parameters online, thereby enabling the Kalman filter to update the model in real time without pre-setting the parameters [12]. In 2024, Tavares et al. proposed an innovative EKF parameter tuning method aimed at improving navigation accuracy in INS/GNSS integrated systems. The authors estimated the process noise covariance matrix Q using ideal trajectory data and inertial error models generated by prior Monte Carlo simulations, and validated the approach on real datasets [13]. In 2025, Liu L. et al. employed the Modified Differential Bees Algorithm (MDBA) to independently optimize the model error covariance Q and measurement noise covariance R in both KF and EKF under scenarios involving unknown inputs [14]. The attention mechanism and EM algorithm used by Jin et al. and the Monte Carlo approach of Tavares Jr. et al. are designed to learn or model integer-order dynamics and are not equipped to navigate the complex interdependencies created by a variable fractional order. Similarly, while Liu et al. use a metaheuristic, their focus on independently optimizing Q and R overlooks the critical coupled effect of α on the entire system.

In summary, the pivotal challenge of parameter selection in FEKF remains a critical, unresolved issue [15]. The choice of the fractional order, α, is not merely a tuning step but a fundamental decision that critically governs the filter’s performance. An inappropriate value can lead to significant degradation through under- or over-filtering, potentially negating the very advantages of the fractional approach. Therefore, optimizing this parameter for a given application is an essential prerequisite to unlocking the full potential of the FEKF.

To address the above issue, this paper proposes the use of Hippopotamus Optimization [16], hereafter referred to as HO, to automatically optimize the fractional-order parameter α in the improved FEKF. HO, introduced in 2024, is a novel swarm intelligence optimization algorithm known for its excellent global search capability and convergence performance, achieving leading results on numerous benchmark test functions. In this study, HO is integrated into the FEKF-based attitude estimation framework to formulate an optimization problem where the attitude estimation error serves as the fitness function, enabling automatic identification of the optimal fractional order α.

Furthermore, to evaluate the effectiveness of the HO algorithm, we compare it with Harris Hawks Optimization (HHO) [17], Genetic Algorithm (GA) [18], Grey Wolf Optimization (GWO) [19], and a new memory mechanism from the machine learning field—HiPPO-LegS [20]. The comparison is conducted from both theoretical and experimental perspectives, analyzing the convergence speed, robustness, and optimality of HO.

## 2. Materials and Methods

### 2.1. Dynamic Model of Attitude Determination

The mathematical formulation of attitude estimation is based on the angular velocity $w={\left(p,q,r\right)}^{T}$ measured by the Inertial Measurement Unit (IMU), where p,q,r denote the angular velocity components about the body-fixed coordinate axes X,Y,Z, respectively. To represent the orientation of the vehicle, Euler angles are commonly employed—namely, roll, pitch, and yaw—which correspond to rotations about the fixed coordinate system’s X,Y,Z axes, respectively [21].

In this study, Euler angles are employed for attitude estimation. The variation in the attitude can be described by the following Equation (1):

(1) $$\left[\begin{array}{l} \dot{\phi }\\ \dot{\theta }\\ \dot{\psi } \end{array}\right]=\left[\begin{array}{ccc} 1 & \sin\phi \tan\theta & \cos\phi \tan\theta \\ 0 & \cos\phi & -\sin\phi \\ 0 & \sin\phi \cos\theta^{-1} & \cos\phi \cos\theta^{-1} \end{array}\right]\left[\begin{array}{c} p\\ q\\ r \end{array}\right]$$

Since the gyroscope in the IMU measures angular velocity, it is necessary to integrate the angular velocity over time in order to obtain the attitude angles:

(2) $$\left\{\begin{array}{l} \phi_{k}=\phi_{k-1}+\dot{\phi }\Delta t\\ \theta_{k}=\theta_{k-1}+\dot{\theta }\Delta t\\ \psi_{k}=\psi_{k-1}+\dot{\psi }\Delta t \end{array}\right.$$

where Δt denotes the sampling time interval, $\phi_{k}$,$\theta_{k}$,$\psi_{k}$ represents the attitude angles at the current time step *k*. $\phi_{k-1}$,$\theta_{k-1}$,$\psi_{k-1}$ represents the attitude angles at the previous time step.

However, direct integration of angular velocity accumulates the gyroscope’s bias drift, causing the attitude estimation error to increase continuously over time. Therefore, practical systems typically combine measurements from accelerometers and magnetometers, employing filtering algorithms such as the Kalman Filter (KF), Extended Kalman Filter (EKF), or complementary filtering to fuse the data and improve the accuracy of attitude estimation.

### 2.2. Fractional-Order Calculus and Fractional-Order Kalman Filtering

#### 2.2.1. Fractional-Order Calculus

The conventional Kalman filter is not inherently suitable for fractional-order systems, which are prevalent in many engineering applications. To enhance the applicability of Kalman filtering in such systems, a novel filtering algorithm known as the Fractional-order Extended Kalman Filter (FEKF) is introduced by integrating fractional-order calculus with the Kalman filter framework. By incorporating fractional-order processing in the error-corrected data update, FEKF significantly improves estimation accuracy.

Using the Grunwald−Letnikov definition, the state-space representation of a fractional-order system can be derived [22]. This leads to a discrete-time fractional-order state-space expression, which is defined as follows:

(3) DαGLf(t)=limh→01hα∑j=0∞(−1)jαjf(t−jh)

In the equation, α represents the order of the fractional derivative; t denotes the sampling interval; and $\left(\begin{array}{c} \alpha \\ j \end{array}\right)$ is defined as the binomial coefficient as follows:

(4) $$\left(\begin{array}{c} \alpha \\ j \end{array}\right)=\left\{\begin{array}{cc} 1 & j=0\\ \frac{\alpha (\alpha -1)(\alpha -2)\cdots (\alpha -j+1)}{j!} & j>0 \end{array}\right.$$

When α is an integer, this expression degenerates to the conventional difference formula. However, when α is a non-integer, the derivative at the current time depends not only on the current value f(t) but also on multiple past values of the function, with their contributions gradually diminishing as k increases. This characteristic endows fractional-order operators with a “long memory” effect, making them more accurate for modeling systems exhibiting fading memory behavior.

#### 2.2.2. Improvements and Applications of FEKF

The fractional-order Kalman filter uses a “predictor–corrector” method. It first predicts the state ${\tilde{x}}_{k}$ based on past data, then corrects that prediction at time t=k using current inertial sensor measurements $y_{k}$. This method models fractional-order non-linear systems as follows:

(5) $$\Delta^{\alpha }{\tilde{x}}_{k+1}=f({\hat{x}}_{k},u_{k})+W_{k}$$

(6) $$y_{k}=h\left(x_{k}\right)+V_{k}$$

In this model, ${\tilde{x}}_{k}$ is the predicted state vector, f represents the non-linear mapping of the state over time, $u_{k}$ is the control input, and the terms $W_{k}$ and $V_{k}$ represent the process noise and measurement noise, respectively. Both are assumed to be zero-mean, uncorrelated Gaussian white noise processes. The objective of this paper is to apply this fractional-order framework to the specific challenge of attitude estimation in environments with strong magnetic interference. In these scenarios, severe electromagnetic interference renders magnetometer data unreliable, which in turn makes the yaw angle unobservable. Therefore, to ensure the filter’s stability and observability, the state vector x and its fractional-order counterpart $\Delta^{\alpha }{\tilde{x}}_{k+1}$ are defined as a five-dimensional vector, composed of the roll angle, the pitch angle, and the three-axis bias errors from the inertial sensors.

(7) $$x_{k}=\left[\begin{array}{c} \begin{array}{c} \theta_{k}\\ \phi_{k}\\ b_{p,k}\\ b_{q,k} \end{array}\\ b_{r,k} \end{array}\right];\Delta^{\alpha }{\tilde{x}}_{k}=\left[\begin{array}{c} \begin{array}{c} \Delta^{\alpha }\theta_{k}\\ \Delta^{\alpha }\phi_{k}\\ \Delta^{\alpha }b_{p,k}\\ \Delta^{\alpha }b_{q,k} \end{array}\\ \Delta^{\alpha }b_{r,k} \end{array}\right]$$

Here, ($b_{p},b_{q},b_{r}$) represent the three-axis gyroscope biases.

The measurement function $h(x_{k})$ maps the five-dimensional state vector to the two-dimensional measurement space. For this linear model, it is defined as $h(x_{k})={\left[\begin{array}{cc} \theta_{k} & \phi_{k} \end{array}\right]}^{T}$.

It is essential to clarify these two equations, which describe the complete estimation problem. They consist of two distinct components: the filter’s prediction algorithm (Equation (5)) and the theoretical system measurement model (Equation (6)).

In the filter prediction model (Equation (5)), the goal is to generate the a priori (predicted) state estimate ${\tilde{x}}_{k+1}$. To do this, the filter must use the best available information from the previous step, which is the a posteriori (corrected) state estimate ${\hat{x}}_{k}$. Using ${\hat{x}}_{k}$ as the input to the nonlinear function f(⋅) is the standard and correct formulation for the prediction step of a recursive filter.

In the theoretical measurement model (Equation (6)), the equation describes how the true, unobservable state $x_{k}$ relates to the actual sensor measurement $y_{k}$, which is corrupted by measurement noise $V_{k}$.

This combined formulation clarifies the distinct roles of the true state ($x_{k}$), the corrected estimate (${\hat{x}}_{k}$), and the predicted estimate (${\tilde{x}}_{k+1}$) within the filter’s operation.

In FEFK, since fractional-order corrections are applied in the fractional-order prediction part, corresponding adjustments are also necessary.

(8) $$F_{k-1}={\left[\frac{\partial f(x,u_{k-1})}{\partial x}\right]}_{x={\hat{x}}_{k-1}}$$

The partial derivatives describe the sensitivity of the system states (roll, pitch angles) to themselves and other state variables. Traditional methods typically neglect the influence of the time step dt on the Jacobian matrix due to system nonlinearity. In this study, by multiplying the above Taylor expansion by the time step dt, a correction term for the state transition Jacobian matrix is obtained:

(9) $$A_{j}=I+F_{k-1}\cdot dt$$

Here, I denotes the identity matrix. This study conducts a detailed analysis of the nonlinear dynamic variations in the roll, pitch, and yaw angles, calculating the sensitivity partial derivatives among the state variables. By capturing cross-coupling effects and extending the computation of each factor’s partial derivatives to include interactions with other factors, the dynamic interactions among variables in the nonlinear system can be better described. Through precise calculation of the partial derivatives of the attitude angle state equations and applying this approach to modify the Jacobian matrix, the resulting Jacobian matrix in this work more accurately characterizes the system’s nonlinear dynamic characteristics, especially under large-angle and high-dynamic conditions.

The covariance prediction step is the key stage where the non-Markovian, long-memory characteristics of the fractional-order system are incorporated into the FEKF algorithm. Unlike the standard EKF, the FEKF computes the a priori (predicted) error covariance $P_{k|k-1}$ by adding a fractional-order covariance correction term $P_{k}^{frac}$ to the standard prediction. This term quantifies the accumulated contribution of historical state uncertainty to the current prediction uncertainty.

First, the fractional coefficient matrix $\gamma_{j}$ is defined. This is a diagonal matrix whose elements are composed of standard binomial coefficients corresponding to each component of our five-dimensional state vector $x={\left[\theta ,\phi ,b_{p},b_{q},b_{r}\right]}^{T}$:

(10) $$\gamma_{j}=dig\left(\begin{array}{cccc} \begin{array}{cc} \left(\begin{array}{c} \alpha_{\theta }\\ j \end{array}\right) & \left(\begin{array}{c} \alpha_{\phi }\\ j \end{array}\right) \end{array} & \left(\begin{array}{c} \alpha_{bp}\\ j \end{array}\right) & \left(\begin{array}{c} \alpha_{bq}\\ j \end{array}\right) & \left(\begin{array}{c} \alpha_{br}\\ j \end{array}\right) \end{array}\right)$$

where $\left(\begin{array}{c} \alpha \\ j \end{array}\right)$ is the standard binomial coefficient as defined in Equation (4), and $\alpha_{\theta },\;\alpha_{\phi },\;\alpha_{bp},\;\alpha_{bq},\;\alpha_{br}$ are the fractional orders corresponding to each of the five states, which are to be optimized.

Next, based on the Grünwald–Letnikov definition, the fractional-order covariance correction term $P_{k}^{frac}$ is computed as follows:

(11) $$P_{k}^{frac}=\sum_{j=2}^{\min(k,L)}\gamma_{j}P_{k-j|k-j}\gamma_{j}^{T}$$

Here, L is the length of the historical window, limiting the memory range of the calculation; and $P_{k-j|k-j}$ is the a posteriori (corrected) error covariance matrix from j steps in the past. This summation (starting from j=2) reflects the influence of historical covariance information, weighted by the fractional coefficients $\gamma_{j}$, on the current predicted covariance.

Finally, this correction term is added to the standard EKF covariance prediction formula to obtain the FEKF’s a priori error covariance matrix $P_{k|k-1}$:

(12) $$P_{k|k-1}=A_{k-1}P_{k-1|k-1}A_{k-1}^{T}+Q_{k}+P_{k}^{frac}$$

where $A_{k-1}$ is the linearized state transition matrix defined by Equation (9), $P_{k-1|k-1}$ is the a posteriori error covariance matrix from the previous step, and $Q_{k}$ is the process noise covariance matrix. Here, *Q* represents the process noise covariance matrix.

In this manner, the FEKF’s covariance prediction explicitly incorporates the historical memory effect inherent in the fractional-order model ($Q_{k}$), in addition to the uncertainty from the current state transition ($A_{k-1}P_{k-1|k-1}A_{k-1}^{T}$) and the process noise ($Q_{k}$), thereby potentially achieving more accurate estimation in systems exhibiting long-memory characteristics.

Measurement Update Stage: In the conventional FEKF, the measurement residual is calculated as:

(13) $$r_{k}=y_{k}-h({\tilde{x}}_{k})$$

In this study, the introduction of fractional-order operators aims to create a filter that is structurally consistent in its handling of historical information. Specifically, the conventional EKF and FEKF update step is designed for integer-order Markovian systems. This creates a structural inconsistency with our process model Equation (5), which explicitly uses a fractional-order term $\Delta^{\alpha }\tilde{x}$ to capture the system’s non-Markovian, long-memory dynamics.

To address this mismatch and align the update mechanism with the system’s fractional-order nature, this paper employs a sliding window approach that accumulates residuals from the past N time steps to construct a fractional-order residual:

(14) $${\tilde{r}}_{k}=\sum_{i=0}^{N}w_{i}r_{k-i}$$

where

(15) $$w_{i}={\left(-1\right)}^{i}\left(\begin{array}{c} \alpha \\ i \end{array}\right)$$

By updating the Kalman gain using ${\tilde{r}}_{k}$ instead of a single $r_{k}$, when α→1 and N=0 hold, the weights $w_{0}=1$ apply while others $w_{i}=0$ cause the FEFK to degenerate into the standard EKF.

The measurement function h(⋅) from Equation (6) is linearized by calculating its Jacobian matrix $H_{k}$ at the predicted state estimate ${\hat{x}}_{k|k-1}$.

(16) $$H_{k}=\frac{\partial h(x)}{\partial x}{|}_{x={\tilde{x}}_{k}}$$

The Kalman gain $K_{k}$ is:

(17) Kk=Pk|k−1Hk(HkPk|k−1Hk+TR)−1T

Here, *R* represents the measurement noise covariance matrix.

By incorporating the aggregated residual ${\tilde{r}}_{k}$ into the FEFK update formula, we obtain:

(18) $${\hat{x}}_{k|k}={\tilde{x}}_{k}+K_{k}{\tilde{r}}_{k}$$

The covariance is calculated using the traditional FEFK method as follows:

(19) $$P_{k|k}=(I-K_{k}H_{k})P_{k|k-1}$$

The selection of the fractional-order parameter α significantly impacts the filtering performance. Therefore, it is necessary to optimize the fractional-order parameter for specific applications. Accordingly, this paper proposes a method based on the HO algorithm to optimize the fractional-order parameter α in the FEFK.

### 2.3. Principle of the HO Algorithm

The HO algorithm is a swarm intelligence optimization method that abstracts the behavioral patterns of hippopotamuses into a mathematical model.

Stage 1: Hippopotamus Position Updating. In the HO algorithm, the solution with the best fitness in the current population is designated as the dominant hippopotamus position, denoted as $D_{hppo}$. During this phase, a subset of the hippopotamuses (candidate solutions) attempts to move closer to the dominant individual to improve the overall quality of the population. The mathematical formulation of this behavior is as follows:

(20) $$x_{i,j}^{new}=x_{i,j}+y_{1}(D_{hppo}-I_{1}x_{i,j})$$

where $x_{i,j}$ represents the current value of the i−th candidate solution in the j−th dimension; $x_{i,j}^{new}$ is the updated value; $y_{1}\in \left[0,1\right]$ is a uniformly distributed random number; $I_{1}\in \left\{1,2\right\}$ is a random integer. When $I_{1}=1$, the increment is:

(21) $$y_{1}(D_{hppo}-x_{i,j})$$

The candidate solution takes a step toward the optimal solution. When $I_{1}=2$, the increment is:

(22) $$y_{1}(D_{hppo}-2x_{i,j})$$

This mechanism enables candidate solutions to randomly drift around the optimal solution. Such a strategy allows some candidates to move away from the current solution, maintaining diversity within the population.

Phase 2: Hippopotamus Defending Against Predators. The HO algorithm introduces the concept of a virtual predator. In each iteration, a “predator” solution Predator is randomly generated. Its position is determined by uniform random sampling within the search space, which enhances the algorithm’s exploration capability by encouraging candidate solutions to escape from potentially suboptimal regions. The position of the predator is mathematically expressed as:

(23) $$P_{j}^{pred}=lb_{j}+r\left(ub_{j}-lb_{j}\right),j=1,2\cdots m$$

where $lb_{j}$ and $ub_{j}$ are the lower and upper bounds of the j−th variable, respectively, and r is a uniformly distributed random number within the range $\left[0,\;1\right]$, The role of is to introduce randomness, acting as the engine for the algorithm’s unpredictability.

This mechanism is mathematically represented by comparing the fitness of the predator, denoted as $F_{\mathrm{Pr}edator}$, with the fitness of the hippo itself, denoted as $F_{i}$, and then applying different position update formulas accordingly. Meanwhile, the distance between the two is defined as:

(24) $$\vec{D}=\left|Predator-x_{i}\right|$$

To simulate the escape behavior of a hippopotamus, the HO algorithm introduces a Lévy random vector RL to generate sudden jumps [23]. When evasion is required, this vector is added to the current position. The mathematical formulation is expressed as follows:

(25) $$\Delta x_{Lévy}\left(\vartheta \right)=0.05*\frac{w\sigma_{w}}{{\left|v\right|}^{\frac{1}{\vartheta }}}$$

If $F(\alpha^{\mathrm{pred}})<F_{i}$, The update formula is given by:

(26) $$x_{i}^{R}=RL⊕P^{pred}+\frac{f}{c-d\cos(2\pi g)}\cdot \frac{1}{D_{i}}$$

When $F_{\mathrm{Predator}}>F_{i}$, Otherwise, only a slight adjustment is made.

(27) $$x_{i}^{R}=RL⊕P^{pred}+\frac{f}{c-d\cos(2\pi g)}\cdot \frac{1}{2D_{i}+r}$$

Here, c~U(1,1.5), d~U(2,3), and g~U(−1,1).Within the algorithm’s update mechanism, the parameters g, c, and d work in synergy to dynamically regulate the search behavior. The role of g is to dynamically adjust the search step size to balance global exploration and local exploitation as the optimization progresses. The influence of c is more pronounced, as it directly controls the intensity of the entire “predator-prey” interaction, effectively managing the algorithm’s strategic shift from an exploratory to an exploitative phase over time. Finally, the role of d is to introduce directional uncertainty into the algorithm’s movement, which provides a more complex random perturbation mechanism than simply adjusting the step size, thereby enhancing its ability to escape from local optima.

Stage 3: Escape Phase. Centered at the current solution, the radius decreases following the iteration process. The candidate new position $x_{i,j}^{E}$ is obtained by random sampling within this local range.

(28) $$x_{i,j}^{E}=x_{i,j}+r\left(lb_{j}^{local}+s_{1}\left(ub_{j}^{local}-lb_{j}^{local}\right)\right)$$

where $s_{1}$ is a random factor that can take one of the following three values:

(29) $$s_{1}=\left\{\begin{array}{cc} \begin{array}{c} 2r_{1}-1\\ r_{2}\\ r_{3} \end{array} & \begin{array}{c} r_{1}∼U(0,1)\\ r_{2}∼N(0,1)\\ r_{3}∼U(0,1) \end{array} \end{array}\right.$$

In each iteration, the algorithm randomly selects one of the above strategies to generate $x_{i,j}^{E}$, thus assigning a corresponding value to $s_{1}$. In this way, the local candidate position may lie on either side of the current solution ($r_{1}$), be biased toward a specific direction ($r_{3}$), or be located near a high-probability region ($r_{2}$). This mechanism increases the likelihood of discovering a better local optimum. The newly generated position is then compared with the original one, and if the fitness improves, the new position is accepted; otherwise, the original solution is retained.

### 2.4. Problem Formulation and Methodology

Problem Formulation: To apply the HO algorithm for parameter optimization within the FEFK attitude estimation framework, it is essential first to define the objective function of the optimization problem. We select the attitude estimation error of the filter over a given time interval as the optimization target. The fitness function is constructed based on the error between the ground truth attitude $x_{true}(k)$ and the FEFK estimated value. The fitness function formulated in this study is:

(30) $$J(\alpha )=\frac{1}{N}\sum_{k=1}^{N}{\left\|\hat{x}(k;\alpha )-x_{true}(k)\right\|}^{2}$$

where $\hat{x}(k;\alpha )$ denotes the attitude estimate obtained by running the FEFK filter up to step k with parameter α. The optimization objective is to find α that minimizes J(α) which corresponds to significantly reducing the attitude estimation error. This problem is typically challenging to solve analytically via differentiation, since J(α) is a complex function derived from nonlinear filtering. However, stochastic optimization algorithms such as the Hippo Optimization (HO) algorithm can be employed to iteratively test different values of α, progressively approaching the optimal parameter.

Parameter Settings: The population size of the HO algorithm is set to N, with the search range for α defined as α∈[0,3]. The range of α should not be too large, as an excessively wide range may diminish the fractional-order memory advantage. Within this range, N initial candidate solutions $\alpha_{i}$. are generated uniformly at random. Additionally, the maximum number of iterations T and other control parameters of the HO algorithm are specified. Other control parameters of the HO algorithm, such as the chasing coefficient and the gravity coefficient, were adopted directly from the original publication by Abdollahzadeh et al. [16]. This decision was made because the source paper demonstrated that these default values provide a robust balance between exploration and exploitation across a wide range of benchmark functions. Adopting these well-vetted parameters ensures a fair baseline for evaluating the algorithm’s performance without introducing confounding variables from parameter meta-optimization.

Implementation Method: For each candidate $\alpha_{i}$, compute the corresponding $J(\alpha_{i})$ using Equation (30) as the fitness value $F_{i}$. Record the best solution $\alpha_{\mathrm{best}}$ and its associated error $F_{\mathrm{best}}$ within the current population, which corresponds to the “leader” hippopotamus $D_{hppo}$.

During the iteration process from t=1 to T, the population solutions are updated according to the three-phase behavioral rules of the HO algorithm. The first half of the population individuals are updated using Equation (20):

(31) $$\alpha_{i,j}^{new}=\alpha_{i,j}+y_{1}(D_{hppo}-I_{1}\alpha_{i,j})$$

This step uses the current best solution $\alpha_{\mathrm{best}}$ and random perturbations to adjust the value of $\alpha_{i}$. The variation of $\alpha_{i}$ can be constrained within the defined domain; for example, if the updated α exceeds the range α∈[0, 3], truncation or clipping is applied. This approach enables a subset of solutions to converge toward the optimum while maintaining sufficient randomness to prevent premature convergence.

Boundary Handling: During the optimization process, it is possible for a candidate solution $\alpha_{i}$ to be updated to a value outside the defined search space. To handle such events, a truncation (or clipping) method is applied. Specifically, if an updated $\alpha_{i}$ exceeds the upper bound (3), it is reset to 3. If it falls below the lower bound (0), it is reset to 0. This deterministic approach was chosen for its simplicity and effectiveness in ensuring all solutions remain within the valid and physically meaningful search domain at all times.

To simulate predator-induced behavior, a virtual predator position $\alpha^{pred}$ is first randomly generated using Equation (23), sampling uniformly within the defined interval. Then, for the latter half of the population, Equation (24) is applied to calculate their distance from $\alpha^{pred}$.

(32) $$\vec{D}=\left|\alpha^{pred}-\alpha_{i}\right|$$

According to their fitness comparison, the values of $\alpha_{i}$ are adjusted. Specifically, if $F(\alpha^{pred})<F_{i}$, then $\alpha_{i}$ is significantly shifted toward $\alpha^{pred}$, as described by Equation (26):

(33) $$\alpha_{i}^{R}=RL+P^{pred}+\frac{f}{c-d\cos(2\pi g)}\cdot \frac{1}{D_{i}}$$

Otherwise, a small adjustment toward $\alpha^{pred}$ is performed according to Equation (27):

(34) $$\alpha_{i}^{R}=RL+P^{pred}+\frac{f}{c-d\cos(2\pi g)}\cdot \frac{1}{2D_{i}+r}$$

This mechanism allows some candidate solutions to be influenced by a randomly generated position. When this random position represents a more optimal direction, the individual can escape from its current local region, thereby accelerating global exploration.

A local random search is applied to all individuals in the population. For each candidate $\alpha_{i}$, a specific neighborhood range is defined. The perturbation factor $s_{1}$, generated from three distinct distributions as described in Equation (29), ensures that the local search can either perform fine-grained exploration around the current solution or jump toward the edge of the neighborhood. The fitness of the newly generated position, $F(\alpha_{i}^{E})$, is evaluated. If the new candidate yields a better fitness, $\alpha_{i}^{E}$, replaces the original $\alpha_{i}$; otherwise, the original solution is retained. This strategy enables each individual to leverage local information for self-improvement, enhancing the exploitation ability of the algorithm in the later stages of the optimization process.

After each round of the above updates, the fitness of the affected individuals is re-evaluated, and $\alpha_{\mathrm{best}}$ along with $F_{\mathrm{best}}$ are updated. If a superior solution emerges, it replaces the current leader. To reduce computational overhead, fitness evaluations are typically deferred during the updates in Stages 1 and 2; the objective function J is recalculated by re-running the FEKF only during Stage 3, when a new solution is accepted, in order to assess its corresponding fitness F.

**Figure 1:**
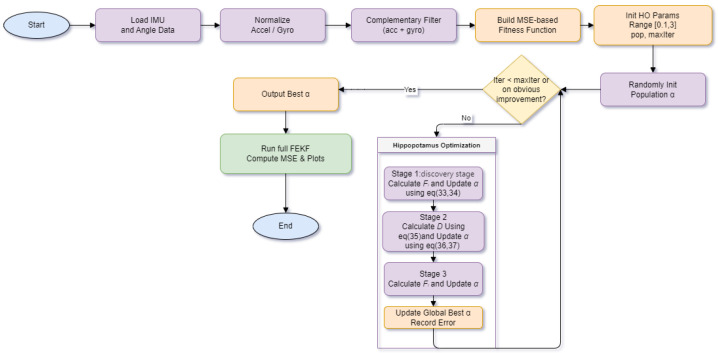
System Flowchart.

Termination Criterion: The iterative process terminates when either the maximum number of iterations is reached or no significant improvement is observed over multiple generations. The best parameter $\alpha_{\mathrm{best}}$ recorded up to that point is output as the optimized result, along with the corresponding minimum estimation error $F_{\mathrm{best}}$. At this stage, the optimal fractional order for the FEKF under the given test scenario is effectively determined, see Figure 1.

## 3. Results

The experimental data utilized in this study include both a publicly available dataset provided by Professor Yan Gongmin’s laboratory [24] and real attitude measurements obtained using a triaxial inertial navigation testing system and flight attitude simulation turntable developed by Beijing JunDa TengFei Technology Co., Ltd., Beijing, China The experiments were conducted in two phases: initially using the open-source dataset, followed by tests with self-collected data.

The following Table 1 are the models of the software and hardware we used:

The improved FEKF with a fixed fractional order was compared against traditional fixed-parameter FEKF, accelerometer-based estimation, gyroscope integration, KF, EKF. Subsequently, the improved FEKF was combined with several intelligent optimization algorithms, and the resulting attitude estimation performance—measured in terms of MSE against ground-truth attitudes—was compared across HO-FEKF, HHO-FEKF, GWO-FEKF, GA-FEKF, and HiPPO-LegS-FEKF.

The experimental results demonstrate that HO-FEKF achieves superior performance in terms of computational cost and estimation accuracy. Moreover, its efficiency and robustness make it suitable for practical deployment in real-world applications.

**Figure 2:**
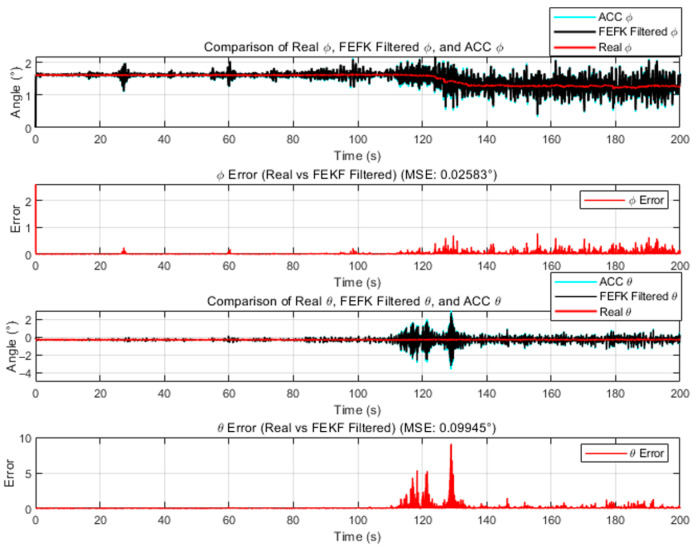
Comparison Between Traditional FEKF with α=1.8 and Truth Attitude Angles.

**Figure 3:**
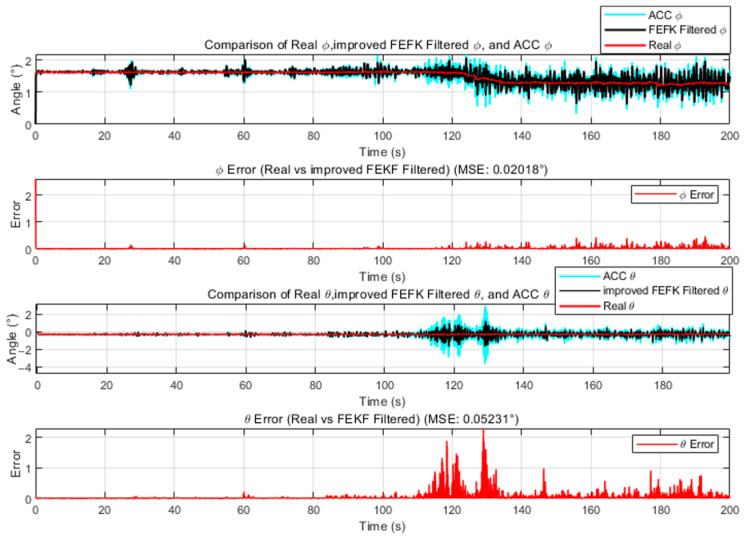
Comparison Between Improved FEKF with α=1.8 and Truth Attitude Angles.

We fix the fractional order of the FEKF at α=1.8. The mean squared errors (MSE) of the fixed-parameter traditional FKEF and the fixed-parameter improved FKEF are shown in Figure 2 and Figure 3, respectively. According to the results, when α=1.8 the fixed-parameter traditional FKEF achieves $MSE_{\phi }=0.02583,MSE_{\theta }=0.09945$. In comparison, the fixed-parameter improved FKEF attains $MSE_{\phi }=0.02018$ $MSE_{\theta }=0.05231$. These results indicate that the FEKF enhanced by the method proposed in this paper achieves higher accuracy than the traditional FEKF.

**Figure 4:**
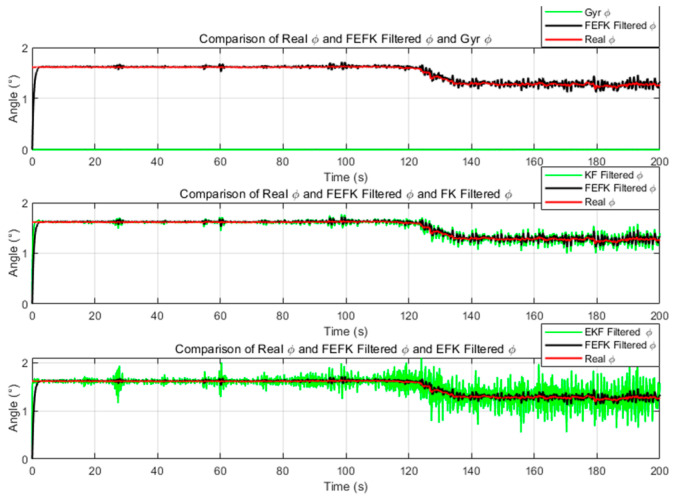
Roll Angle Error Comparison Between Improved FEKF with α=1.8, Gyroscope, KF, EKF.

**Figure 5:**
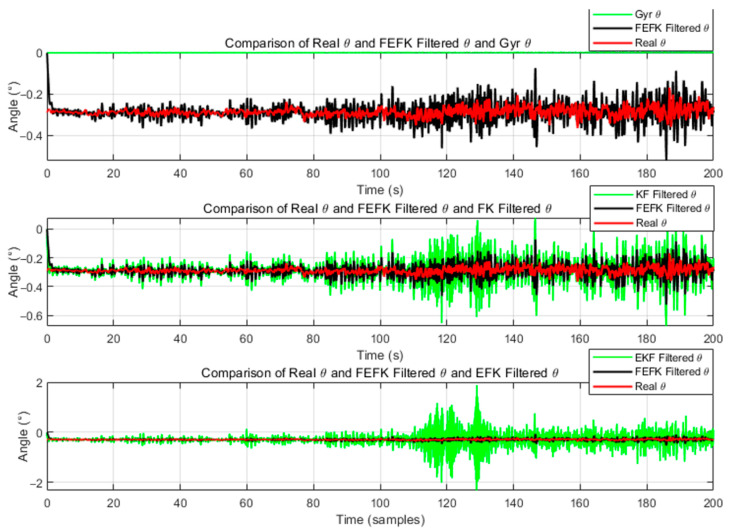
Pitch Angle Error Comparison Between Improved FEKF with α=1.8, Gyroscope, KF EKF.

For the fixed-parameter improved FKEF at α=1.8, the attitude estimation errors relative to the true angles—compared with those from the gyroscope, KF and EKF—are illustrated in Figure 4 and Figure 5.

As shown in Figure 4 and Figure 5, the improved FEKF proposed in this study clearly outperforms the gyroscope-based method, KF, and EKF in estimating the attitude angles.

**Figure 6:**
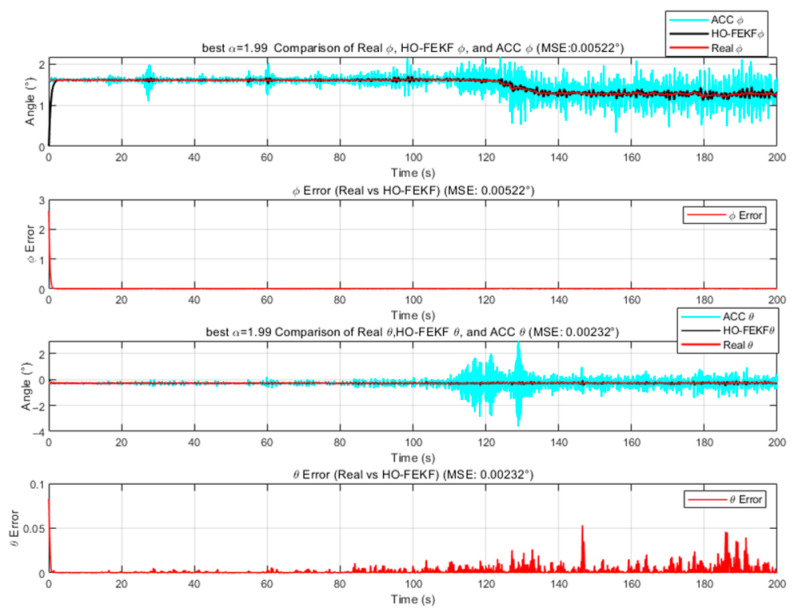
Estimation Results of HO-FEKF.

**Figure 7:**
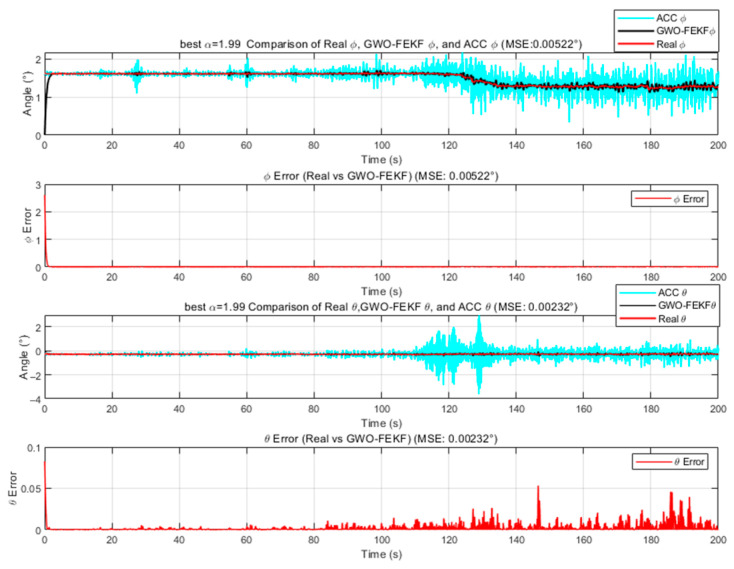
Estimation Results of GWO-FEKF.

**Figure 8:**
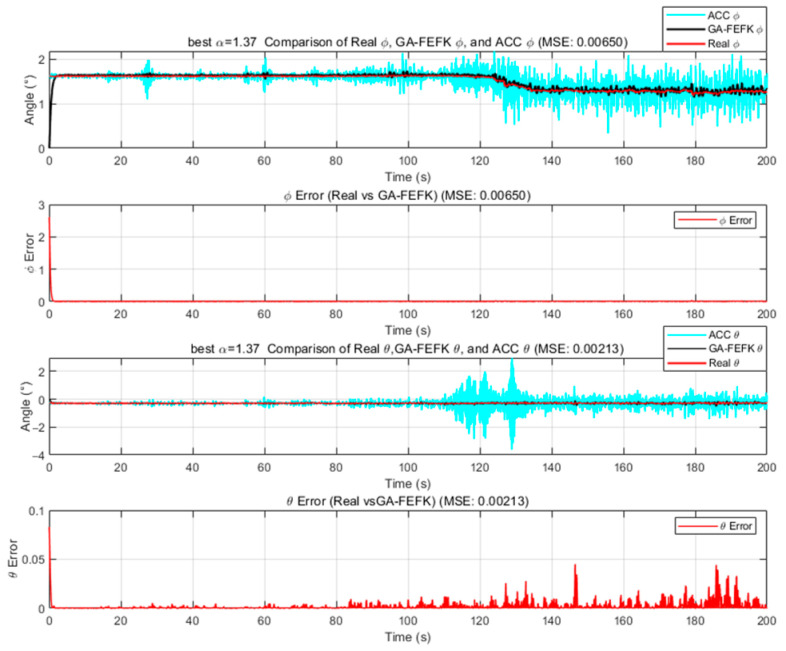
Estimation Results of GA-FEKF.

**Figure 9:**
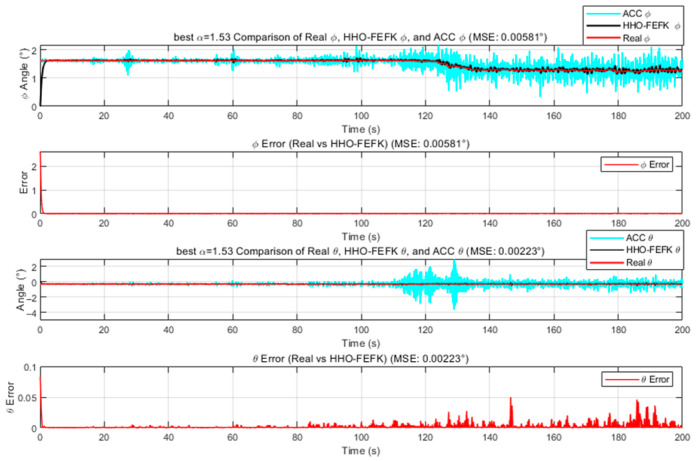
Estimation Results of HHO-FEKF.

**Figure 10:**
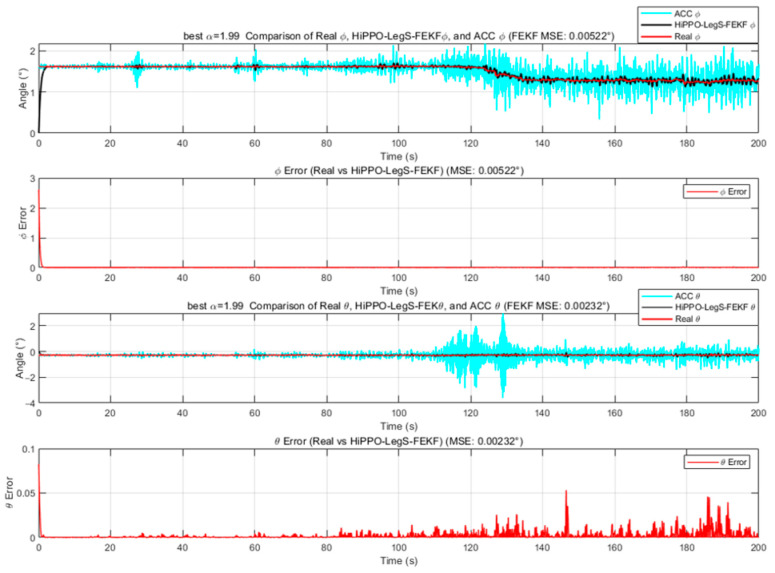
Estimation Results of HiPPO-LegS-FEKF.

We employ the improved FEKF in conjunction with an intelligent algorithm to automatically search for the optimal fractional-order parameter, leading to the proposed HO-FEKF. To demonstrate the superiority of the algorithm, we compare its performance with HHO-FEKF, GWO-FEKF, GA-FEKF, and HiPPO-LegS-FEKF. It is worth noting that GWO-FEKF and GA-FEKF tend to converge to local optima when the population size is set too small. Therefore, to ensure a fair and effective comparison, the population size for these algorithms is set to 30 in this study, see Figure 6, Figure 7, Figure 8, Figure 9 and Figure 10.

The results are presented in Table 2.

To ensure fairness, all algorithms were configured with an identical population size 20 and search space [0, 3]. Furthermore, to maintain consistency with the unit labeling in the figures (which use degrees, °), all error metrics reported in the tables and text are also presented in degrees (°). Furthermore, we applied a unified, two-part termination criterion to all algorithms:

A maximum iteration limit: Set to 30 generations, ensuring no algorithm was allocated a larger computational budget than another.

A common early stopping rule: The run would terminate if the best-found solution did not improve for 10 consecutive generations.

A fine-grained grid search of the parameter space empirically verified that the global optimum for this dataset corresponds to α=1.99 The performance of each algorithm in finding this optimum is detailed in Table 1 and visualized in the convergence plots.

Our proposed HO-FEKF framework successfully identified this global optimum, converging to α=1.99 in approximately six generations. This rapid and accurate convergence can be attributed to the inherent characteristics of the Hippopotamus Optimization algorithm. HO maintains a robust balance between global exploration and local exploitation, but critically, it incorporates a unique mechanism to actively avoid local optima. This feature is particularly valuable because the relationship between the FEKF’s parameters and its performance is highly complex, creating numerous “local optima” that can easily trap standard algorithms. The HO algorithm is specifically designed to overcome this challenge; by identifying and escaping these false solutions, it can effectively navigate the entire search space to locate the true global optimum.

The GWO-FEKF and HiPPO-LegS-FEKF algorithms also eventually found the optimal fractional order. However, their convergence was significantly less efficient. GWO, while a capable global optimizer, follows a simpler social hierarchy-based search pattern that lacks the sophisticated local-optima-avoidance strategy of HO, leading to more generations required to escape suboptimal regions. HiPPO-LegS-FEKF, while accurate, was computationally prohibitive. Its underlying methodology, rooted in continuous-time memory systems, requires complex internal state calculations for every fitness evaluation. This inherent complexity resulted in the longest computation time, raising questions about its practicality for offline tuning tasks where efficiency is a concern.

GA-FEKF converged prematurely to a suboptimal value of α=1.37. This is a classic failure mode for Genetic Algorithms when faced with a complex, multimodal search space. We attribute this to the limited efficacy of its standard crossover and mutation operators in this context [25]. HHO-FEKF converged to a significantly worse local minimum of α=1.53. This algorithm, which models the hunting behavior of hawks, is known for its powerful and often aggressive exploitation phase. While this allows it to converge very quickly (35.66 s), it also makes it highly susceptible to getting trapped. The algorithm’s strategy is to operate on a “greedy” principle: upon discovering a seemingly good solution in the initial stages, it rapidly concentrates all its resources on local optimization. However, this greedy strategy causes it to prematurely lock onto an incorrect region, completely abandoning the global search and thereby missing the opportunity to find the true optimum entirely.

Practically, our findings demonstrate that the full potential of advanced filters like FEKF can only be unlocked through a carefully chosen, automated tuning process. The baseline comparison, where a manually tuned FEKF (α=1.8) yielded a significantly higher error, clearly shows the unreliability of manual tuning. The HO-FEKF framework provides a robust and efficient method to achieve high-accuracy, adaptive attitude estimation, removing the need for expert intuition and laborious trial-and-error, thereby enhancing the practical applicability of fractional-order filtering in real-world scenarios.

**Figure 11:**
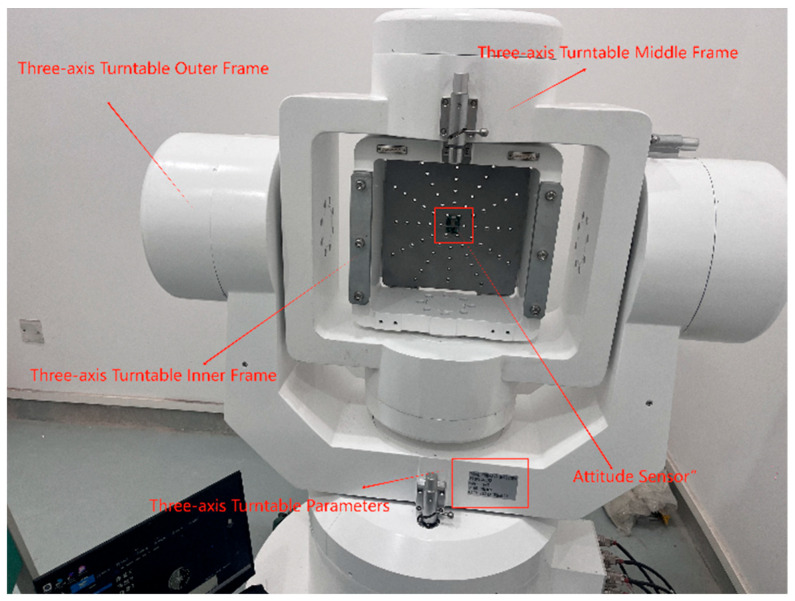
Triaxial Inertial Navigation Test and Flight Attitude Simulation Platform.

To further validate the feasibility of the proposed method, this study employed a three-axis inertial navigation test combined with a flight attitude simulation turntable to conduct algorithm verification. The data measured from the three-axis inertial navigation system and the flight attitude simulation turntable were used as the ground truth for attitude angles, serving as a benchmark for comparative experiments. The testing setup is illustrated in Figure 11.

**Figure 12:**
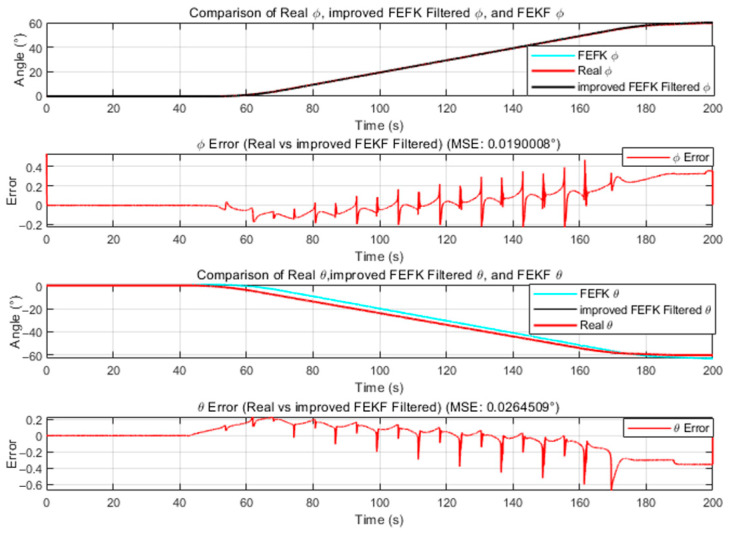
α=1.8 Comparison between the improved FEKF and the traditional FEKF under dynamic conditions.

A three-axis inertial navigation test combined with a flight attitude simulation turntable was conducted, during which both the roll and pitch angles were simultaneously rotated by $60^{{}^{\circ}}$. This setup was designed to evaluate the performance of the improved FEKF under large-amplitude dynamic conditions, in comparison with the traditional FEKF, KF, and EKF. The fractional order for both the traditional and improved FEKF was fixed at α=1.8. The results are presented in Figure 12, where it is evident that the improved FEKF demonstrates superior performance over the traditional FEKF.

**Figure 13:**
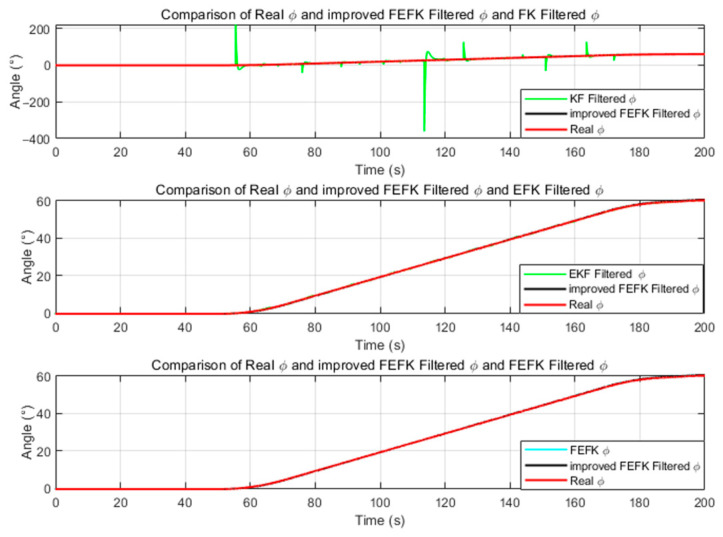
α=1.8 Comparison of the roll estimation under dynamic conditions among the improved FEKF (with), traditional FEKF, KF, and EKF.

**Figure 14:**
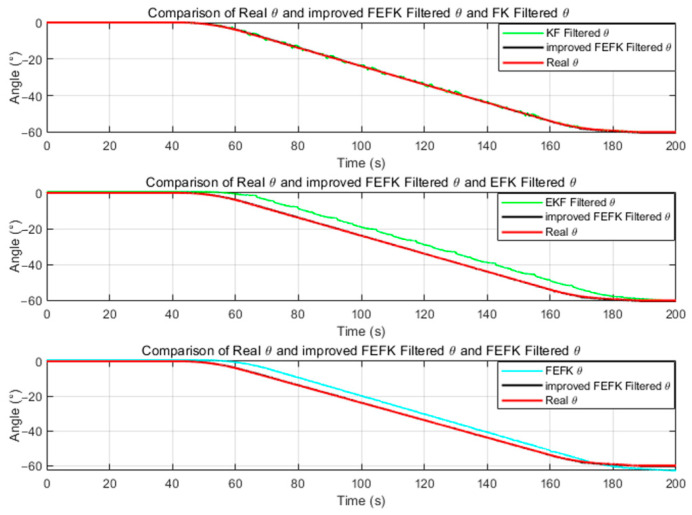
α=1.8 Comparison of the pitch estimation under dynamic conditions among the improved FEKF (with), traditional FEKF, KF, and EKF.

As shown in Figure 13 and Figure 14, the standard KF demonstrates better performance in pitch angle estimation compared to the EKF and traditional FEKF, though it is slightly inferior to the improved FEKF. However, its roll estimation suffers from significant fluctuations and large errors, indicating poor stability. The EKF and traditional FEKF exhibit better results in roll estimation, yet their pitch estimation errors are greater than those of the KF and improved FEKF. Overall, the traditional FEKF outperforms both KF and EKF in combined performance, but it is still clearly inferior to the improved FEKF. These findings indicate that the improved FEKF provides superior performance under large-amplitude dynamic conditions compared to the traditional FEKF, KF, and EKF.

**Figure 15:**
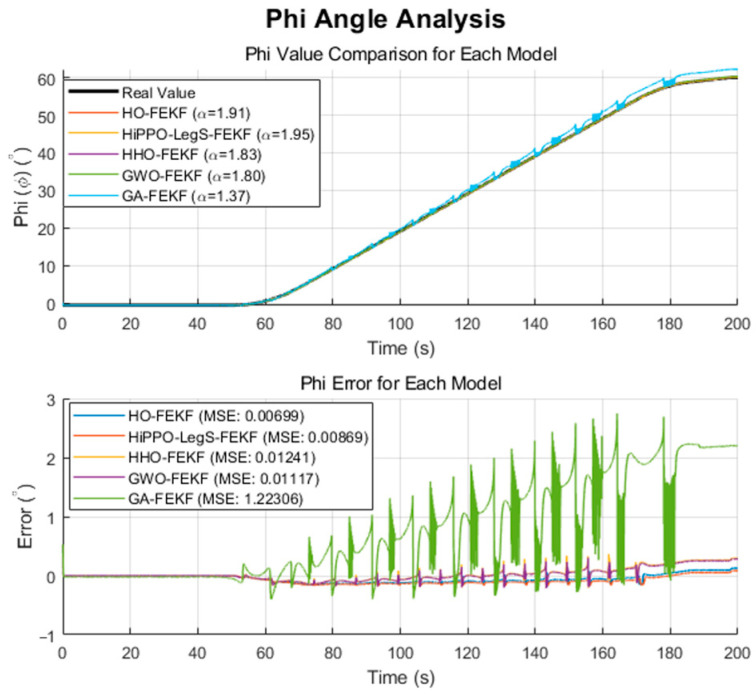
Estimation Results of HO-FEKF.

**Figure 16:**
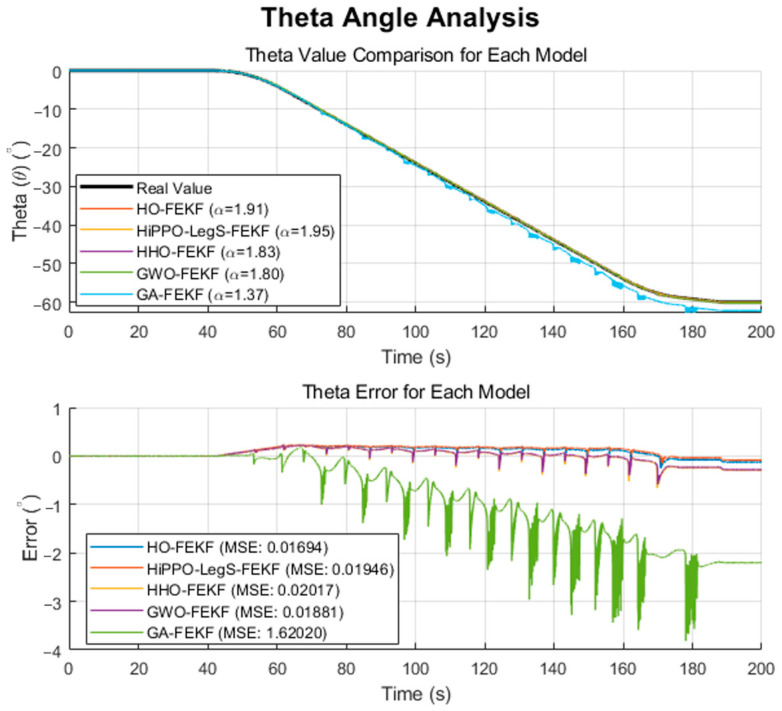
Estimation Results of GWO-FEKF.

The performance comparisons of HO-FEKF, GWO-FEKF, GA-FEKF, HHO-FEKF, and HiPPO-LegS-FEKF under large-angle rotational conditions, in relation to the true attitude angles, are illustrated in Figure 15 and Figure 16.

The results are presented in Table 3.

Through a fine-grained grid search, we verified that α=1.91 is the global optimum for this dataset. Our HO-FEKF not only successfully achieved this target but also demonstrated superior convergence speed and computational efficiency. However, the most revealing discovery came from the challenging test on the large-amplitude, real-world rotational dataset. This scenario acted as a touchstone, exposing the fragility of other competing algorithms in practical applications as HHO-FEKF, GWO-FEKF, GA-FEKF, and HiPPO-LegS-FEKF all failed in this test, becoming trapped in suboptimal local minima.

This collective failure reveals a key insight: the search space for real-world FEKF parameter optimization is far more rugged and deceptive than what is presented by benchmark data. The abnormally high mean squared error exhibited by GA-FEKF, in particular, is rooted in the inherent “premature convergence” defect of the genetic algorithm when handling such problems. Due to its population losing genetic diversity too early, the standard crossover and mutation operators were powerless to drive the solution out of a strong local optimum trap, causing it to converge in the wrong region. Similarly, although HHO has a fast iteration speed, its aggressive exploitation strategy makes it a high-risk option, while the success of GWO and HiPPO-LegS is limited to simpler scenarios, exposing their lack of robustness when facing complex dynamics.

In summary, all evidence indicates that HO-FEKF is not just an incremental improvement in performance but a fundamentally more effective and reliable solution. Its ability to maintain robust global search capabilities even in the most challenging scenarios fully confirms the value of its advanced algorithmic design and its immense practical utility in solving the intractable problem of fractional-order parameter optimization in FEKF.

## 4. Conclusions and Future Scope

The conclusion of this study is not merely that the HO-FEKF algorithm is numerically superior to other methods, but rather, through an in-depth analysis of the experimental results, it reveals the inherent challenges of the fractional-order system parameter optimization problem and demonstrates why the HO algorithm is an effective tool for its solution.

Our core argument is built upon the following key experimental evidence:

The Objective Existence of the Global Optimum and the Precise Convergence of HO-FEKF: Through a fine-grained grid search, we first established α=1.99 as the indisputable global optimum for the public dataset scenario, setting a gold standard for the performance evaluation of all algorithms. Against this benchmark, the experimental results clearly show that the HO-FEKF was the only framework capable of converging to this optimum point stably and efficiently. Its rapid convergence within a minimal number of generations is a direct testament not only to its efficiency but also to its algorithmic robustness.

The “Failure Modes” of Competing Algorithms as Counter-evidence: The failures of other algorithms were not simply poor performance: their specific failure modes provide powerful counter-evidence for our thesis. The GA converged to a suboptimal α=1.91, resulting in a significantly higher root-mean-square error. This precisely exposes the classic defect of “premature convergence” that arises from a premature loss of population diversity when faced with a complex optimization landscape. Even more striking was the Harris Hawks Optimizer (HHO); although it had the fastest per-iteration time, it converged to a distant local trap (α=1.45). This validates that its “greedy” exploitation strategy is high-risk and unreliable when confronted with problems containing deceptive local optima.

The “Stress Test” of Real-World Data as the Definitive Proof: The most convincing evidence was derived from the large-amplitude, real-world rotational dataset. In this experiment, which simulates a true engineering challenge, all competing algorithms—including GWO and HiPPO-LegS, which performed adequately on the simpler benchmark—failed and became trapped in local optima. The singular success of HO-FEKF in this extreme scenario definitively proves that its advanced mechanism for local optima avoidance is not merely an enhancement but a necessary condition for ensuring system reliability in practical applications.

In summary, the conclusion drawn directly from this body of evidence is that the value of the HO-FEKF framework extends far beyond providing a filter with lower error. Its true contribution lies in successfully solving an optimization problem that is fundamentally intractable for traditional metaheuristic algorithms. Therefore, we have not only proposed a new method but have also, through rigorous comparison and failure analysis, demonstrated why it is the correct tool for the FEKF parameter optimization challenge, thereby genuinely bridging the gap from fractional-order filtering theory to high-performance, high-reliability practical application.

Building upon the successful validation of the HO-FEKF framework, several promising and high-impact avenues for future research are evident:

Unified Multi-Parameter Co-optimization: The current work focused on the critical parameter α. A significant and logical next step is to expand the HO framework to perform a simultaneous, co-optimization of the complete parameter set $\left\{\begin{array}{ccc} \alpha & Q & R \end{array}\right\}$, where Q and R are the process and measurement noise covariance matrices. This would holistically address the strong coupling effects between these parameters and could unlock further performance enhancements. Extension to Broader Application Domains: The demonstrated success of the HO-FEKF in attitude estimation strongly suggests its potential applicability to other complex state estimation problems where fractional-order dynamics are likely present. Future research could explore its utility in diverse fields such as robotics, biomedical signal processing, and financial time-series forecasting.

## Figures and Tables

**Table 1:** Introduction to Software and Hardware Specifications.

Hardware	Software
CPU: [Intel Core i7-12700K @ 4.2 GHz] (Intel: Santa Clara, CA, USA)	Operating System: [Windows 11 64-bit]
RAM: [32 GB DDR5 @ 4800 MHz]	Environment: [MATLAB R2023b]
IMU Model: [JY61P] (100Hz)	Programming Language: MATLAB

**Table 2:** α Optimization Performance Comparison.

Algorithm	SPEED	TIME	$S\left(n\right)$	α	$MSE_{\phi }$	$MSE_{\theta }$
FEKF	/	/	O(M)	1.8	0.02583	0.09945
Improved FEKF	/	/	O(M)	1.8	0.02018	0.05231
GWO-FEKF	10	141.38S	O(600M)	**1.99**	**0.0052** **2**	0.00232
GA-FEKF	15	568.42S	O(600M)	1.37	0.00650	**0.00213**
HHO-FEKF	**5**	**35.66S**	**O(20M)**	1.53	0.00581	0.00223
HiPPO-LegS-FEKF	10	105.75S	O(100M)	**1.99**	0.00522	0.00232
**OUR**	6	39.15S	**O(20M)**	**1.99**	**0.00522**	0.00232

**Table 3:** α Optimization Performance Comparison.

Algorithm	SPEED	TIME	$S\left(n\right)$	α	$MSE_{\phi }$	$MSE_{\theta }$
GWO-FEKF	15	198.48s	O(600M)	1.80	0.01117	0.01881
GA-FEKF	20	368.42s	O(600M)	1.37	1.22306	1.62020
HHO-FEKF	9	46.66s	**O(20M)**	1.83	0.01241	0.02017
HiPPO-LegS-FEKF	13	135.37s	O(100M)	1.95	0.00869	0.01946
**OUR**	**8**	**46.22s**	**O(20M)**	**1.91**	**0.00699**	**0.01694**

## Data Availability

Since our data is confidential to the funder and cannot be provided, the public dataset is available at the following URL: https://psins.org.cn/newsinfo/4445782.html (accessed on 18 June 2025).
